# Supplementary material for: Marangoni-driven flower-like patterning of an evaporating drop spreading on a liquid substrate
Source: Nat Commun. 2018 Feb 26;9:820. doi: 10.1038/s41467-018-03201-3 (PMC5827038; doi:10.1038/s41467-018-03201-3)
Supplement: Supplementary file 1 — Supplementary Information [file 41467_2018_3201_MOESM1_ESM.pdf]

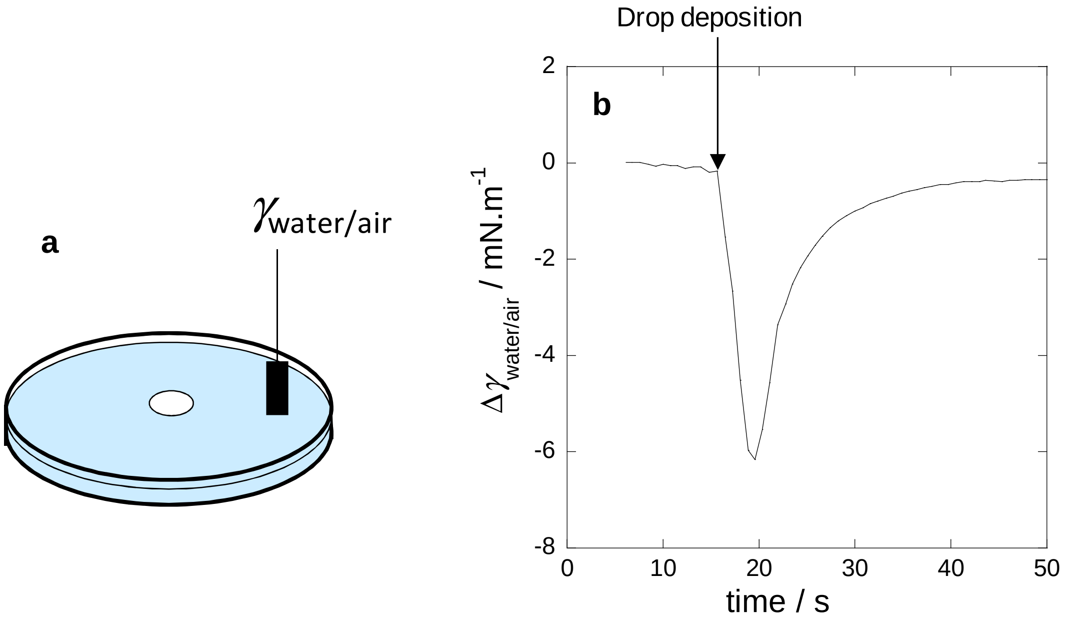

Supplementary Figure 1: **Experiment revealing the surface activity of DCM on water**. **a** sketch of the measurement setup, with the Wilhelmy plate inserted 3cm away from the centre of the Petri dish; **b** evolution of the water/air surface tension after a 25 μL drop has been released, revealing a fast 6 $mN.m^{-1}$ decrease of $\gamma_{water/air}$. Beyond that point, the drop fades away and the surface tension gradually recovers its initial value.

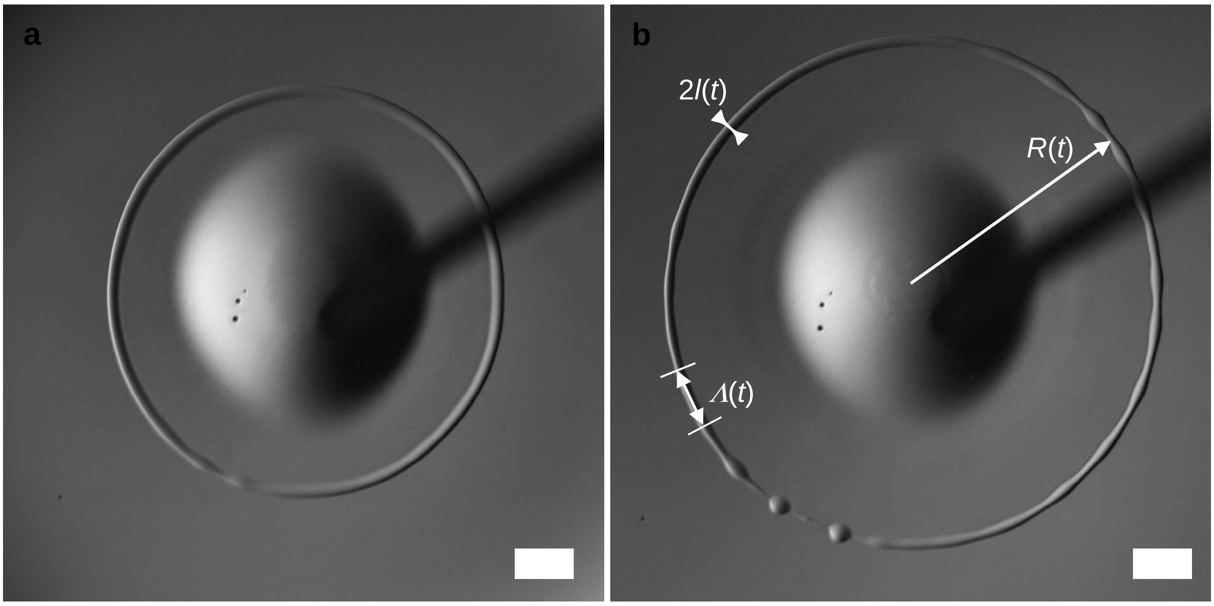

Supplementary Figure 2: Evolution of rim varicosities. **a** rim geometry at time t_1; **b** at time $t_2 = t_1 + 14.86$ ms. Values of the most amplified wavelength Λ were obtained by determining the distance between the two most pronounced bulges in **a**, and between the two bulges at the left of the two drops in **b**; the (larger) wavelength comprised between the two drops in **b** was excluded because it is already influenced by nonlinear mechanisms, as testified by the presence of a satellite droplet in between the two drops.

	Λ/l (t=t_0)	Λ/l (t=t_1)
theory, $T_\gamma/T_s \to 0$, $T_{\mu o}/T_s \to 0$	9.01	9.94
theory, $T_\gamma/T_s \to 0$, $T_{\mu o}/T_s = 1$	6.65	7.53
experiments, $T_\gamma/T_s \approx 0.0073$, $T_{\mu o}/T_s = 0.91$	—	7.4

Supplementary Table 1: Theoretical prediction and experimental determination of the most amplified disturbance during pulsation P_1. The most amplified wavelength, Λ, is normalized by the rim half-thickness, l. Experimental values of time ratios T_γ/T_s and $T_{\mu o}/T_s$ refer to time t_1.
