# Supplementary material for: Marangoni-driven flower-like patterning of an evaporating drop spreading on a liquid substrate
Source: Nat Commun. 2018 Feb 26;9:820. doi: 10.1038/s41467-018-03201-3 (PMC5827038; doi:10.1038/s41467-018-03201-3)
Supplement: Supplementary file 3 — Description of Additional Supplementary Files [file 41467_2018_3201_MOESM3_ESM.pdf]

Description of Additional Supplementary Files

File Name: Supplementary Movie 1
Description: Experiment described in the article. A 5.6 μl dichloromethane drop is deposited onto a 25 ml aqueous phase filling a 7cm Petri dish. A cationic surfactant, cetyltrimethylammonium bromide (CTAB), is initially present in both phases with the same concentration (0.5 mmol.L^{-1}). The sequence is slowed down 20 times.

File Name: Supplementary Movie 2
Description: Magnification of the receding stage during the sequence displayed in Supplementary Movie 1, providing details of the droplets ejection process during film recoil. The sequence is slowed down 200 times.
